# Subtypes I and II of *Ulva prolifera* O.F. Müller: Dominant Green Tide Species in the Southern Yellow Sea and Their Responses to Natural Light and Temperature Conditions

**DOI:** 10.3390/biology14060702

**Published:** 2025-06-15

**Authors:** Shuang Zhao, Jinlin Liu, Zhangyi Xia, Jingyi Sun, Jianheng Zhang, Peimin He

**Affiliations:** 1 Ocean College, Fujian Polytechnic Normal University, Fuqing 350300, China; zhaos@fpnu.edu.cn; 2 Fujian Provincial Key Laboratory of Coastal Basin Environment, Fujian Polytechnic Normal Univeristy, Fuqing 350300, China; 3 State Key Laboratory of Marine Geology, Tongji University, Shanghai 200092, China; 4 State Key Laboratory of Marine Environmental Science, College of Ocean and Earth Sciences, Xiamen University, Xiamen 361102, China; xzy19028@163.com; 5 College of Oceanography and Ecological Science, Shanghai Ocean University, Shanghai 201306, China; sjyyjs2009@163.com (J.S.); jh-zhang@shou.edu.cn (J.Z.)

**Keywords:** *Ulva prolifera*, ecotypic differentiation, morphology, growth, photosynthetic acclimation

## Abstract

This study systematically investigated two ecotypes of *Ulva prolifera*, the dominant species responsible for green tides in the Yellow Sea, classified as Subtype I (strain *I08-1*) and Subtype II (strain *QD-7*). Both subtypes produce positively phototactic biflagellate gametes with oval/pear-shaped morphology but exhibit distinct cellular dimensions. Subtype I gametes demonstrated significantly larger cell sizes, with long and short axes measuring 6.55 μm and 4.62 μm, respectively, compared to Subtype II’s dimensions of 6.46 μm (long axis) and 3.03 μm (short axis). Developmental analysis revealed striking morphological divergence at the 6-day germling stage: Subtype I attained an average length of 1301.14 μm, more than doubling Subtype II’s 562.25 μm. Superior growth kinetics were observed in Subtype I, exhibiting enhanced specific growth rates (SGRs) across multiple parameters—main stem length (8.58% vs. 3.55%), primary branch elongation (19.17% vs. 12.59%), main stem width expansion (17.29% vs. 5.00%), and biomass accumulation (41.90% vs. 40.96% fresh weight). Chlorophyll quantification confirmed significantly higher pigment content in Subtype I. Pre-co-culture photosynthetic profiling demonstrated Subtype I’s superior quantum efficiency (α = 0.077 vs. 0.045) with marked differences in regulated energy dissipation (YNPQ) and non-photochemical quenching (NPQ). Post-co-culture physiological adaptation was evident in Subtype II, showing significant elevation of non-regulated energy dissipation quantum yield (YNO) and eventual surpassing of maximum electron transport rate (ETRmax) compared to Subtype I. These findings establish that *U. prolifera* employs robust photoprotective and thermal adaptation strategies under natural photothermal conditions. Crucially, YNO-based analysis revealed Subtype II’s enhanced high-light protection mechanisms and superior adaptability to intense irradiance environments. This research elucidates ecotype-specific environmental adaptation mechanisms in *U. prolifera*, providing critical insights for optimizing green tide mitigation strategies and advancing ecological understanding of algal bloom dynamics.

## Simple Summary

Since 2007, large-scale green tides have persisted in China, causing adverse effects on the ecological environment, aquaculture, coastal landscapes, and the water sports industry. To clarify the morphological, growth, and photosynthetic differences in the dominant green tide species *Ulva prolifera* across outbreak years, this study compared samples from 2008 and 2021 through molecular identification and comparative culture observations. Results indicate that the dominant species has undergone adaptive changes in environmental tolerance since the initial green tide outbreak. In 2008, seedlings exhibited faster growth rates, greener pigmentation, and more abundant primary branches. In contrast, adult *U. prolifera* populations in 2021 demonstrated higher branching complexity, enhanced environmental adaptability, and accelerated growth rates. These findings suggest that under the combined influence of natural and anthropogenic forces, *U. prolifera* has evolved adaptive traits that likely contribute to the expanding scale of green tide outbreaks. This study deepens our understanding of the environmental adaptation mechanisms in green tide-forming species and provides a scientific foundation for developing prevention strategies and elucidating the occurrence mechanisms of *U. prolifera* blooms.

## 1. Introduction

Since 2007, large-scale green tides have persistently occurred along China’s coastlines [1,2,3,4], imposing adverse impacts on the ecological environment, aquaculture, coastal landscapes, and water sports industries [5]. These recurring events have consequently drawn significant attention from the government and relevant authorities. Green tides in this area are primarily driven by multiple factors, including local seawater eutrophication (often linked to organic pollution from anthropogenic sources such as nutrient discharge) [6,7], rising seawater temperatures (associated with global warming) [8,9], and other anthropogenic activities. Upon detaching from substrates, macroalgae such as *U. prolifera* leverage air sac structures within their thalli to form floating populations. The rapid proliferation and sudden aggregation of thalli then give rise to large-scale floating algae mats, an abnormal ecological phenomenon documented in previous studies [10,11,12]. The onset of green tides not only disrupts marine traffic but also triggers oxygen depletion in seawater through algal respiration and decomposition, which can potentially suffocate aquatic organisms and induce secondary marine disasters. Moreover, large expanses of floating green algae often encroach upon coastal aquaculture areas, reducing mariculture yields [11,13] and causing substantial economic losses.

The oxygen consumption, release of toxic substances/odorous gases, and decay of green algae pose multifaceted threats: they endanger human health, poison aquatic organisms, degrade marine ecosystems, and disrupt the normal functioning of coastal city tourism [14,15,16]. The decomposition of green algae generates sulfur-containing compounds that exacerbate water quality deterioration and environmental harm [14,15,16]. When macroalgae accumulate, sink, and decompose along coastlines or in open oceans, their decay consumes dissolved oxygen, creating localized hypoxic or anoxic zones [17]. Concurrently, this process releases abundant sulfur compounds and other byproducts, causing severe secondary ecological damage and economic losses. Beyond impeding coastal mariculture production [18,19], green macroalgae directly undermine tourism and economic development in coastal cities [20]. Their decomposition also discharges substantial allelochemicals, sulfides, and nutrients into coastal waters [21]. In fish, shrimp, sea cucumber, and shellfish aquaculture zones, green tide algae reduce primary productivity, indirectly destabilizing both aquaculture operations and the marine ecological environment. During decomposition, green tide algae deplete dissolved oxygen, leading to mass mortality of shellfish due to hypoxia [18,19]. While floating algae mats may benefit herbivorous marine invertebrates and suspension-feeding animals, large benthic deposit-feeders and tide-dependent mobile bivalves suffer negative impacts from green tides [22].

Based on Landsat data, floating *U. prolifera* have been observed in the Southern Yellow Sea since 1999. This phenomenon was first detected by a moderate-resolution imaging spectroradiometer in 2007 [23], yet it was not until 2008 that *U. prolifera* green tides began large-scale outbreaks, raising widespread concern. Since then, annually from April to August, green tides drift northward driven by wind currents [24,25]. Combined with suitable temperatures and abundant nutrients in the Yellow Sea, the green tides persistently expand [26], primarily affecting the coastal waters of Shandong and Jiangsu Provinces. Specifically, annual accumulated *U. prolifera* biomass exceeding 1.5 million tons impacts key areas including Qingdao (Shandong), Rizhao (Shandong), and Lianyungang (Jiangsu). Local governments have implemented a cross-provincial coordination mechanism, allocating over CNY 200 million yearly toward mitigation measures, including early monitoring, mechanical harvesting, and ecological remediation. Consequently, green tide prevention and control have drawn significant research attention [27,28,29,30,31]. Among strategies, source prevention represents the most economical and effective approach [32,33]. Previous studies have identified green tides primarily caused by *Ulva* species, such as *U. prolifera*, *U. flexuosa*, *U. lactuca*, *U. linza*, *U. pertusa*, *U. compressa*, and *U. meridionalis* [34,35,36,37], with *U. prolifera* as the dominant species in the Southern Yellow Sea [38]. Key causative factors include primarily seawater eutrophication, along with suitable temperature, salinity, and light intensity, and the biological traits of *U. prolifera*. Large-scale outbreaks depend on abundant nitrogen and phosphorus [12]. Variations in temperature and light govern *U. prolifera* floating and decay, while high light intensity and optimal temperature extend the high-growth period [39,40,41].

Appropriate light intensity facilitates photosynthesis in *U. prolifera* and promotes its growth, thereby increasing green tide biomass [42]. *Ulva* is an opportunistic genus with strong nutrient absorption capacity, rapid reproduction, high light-energy utilization efficiency, and robust stress resistance [43,44,45]. Furthermore, the air sac structure of *U. prolifera* enables it to float. Thus, green tide formation is not only directly linked to seawater eutrophication [6] but also closely associated with rising spring seawater temperatures [46] and *U. prolifera*’s unique biological traits [47]. As *Ulva* morphology and phenotype vary across growth stages and environmental conditions [3], molecular marker technology plays a critical role in species identification. Zhang et al. [48] utilized internal transcribed spacers (ITS) and 5S ribosomal intergenic spacers to characterize attached and floating green tide algae in Rudong, Jiangsu Province, China. Aquaculture rafts for *Neopyropia yezoensis* in the Jiangsu Shoals provide suitable substrates for *U. prolifera* attachment and germination, thereby enhancing its reproduction [49,50]. To mitigate green tide disasters, the Chinese government has allocated substantial funds and deployed considerable manpower and material resources for source control [28,51].

The dominant species of green tides in the Yellow Sea exhibit population succession patterns and include different *Ulva* strains. During the early stages of green tide outbreaks in the Southern Yellow Sea, the dominant free-floating green algal species are *U. compressa*, *U. linza*, *U. prolifera*, and *U. flexuosa*, which are the same as the dominant green algae on *Neopyropia* rafts. However, only one free-floating alga species, *U. prolifera*, continues to multiply and grow, leading to large-scale green tide outbreaks [52,53]. Seawater-borne green algal propagules, *U. prolifera* attached to *Neopyropia* aquaculture rafts, and the outbreak species *U. prolifera* all belong to the same ecological type [53]. Thus, the widely accepted view is that *U. prolifera* originates from *Neopyropia* aquaculture raft areas in the Southern Yellow Sea. In contrast, a less accepted hypothesis suggests that Yellow Sea green algae may originate from animal aquaculture ponds along the Southern Yellow Sea coast [54,55], the seabed [56], or coastal green algae distribution areas spanning from the south of the Shandong Peninsula to the north of the Yangtze Estuary. Wang et al. [35] revealed that the dominant species of the green tide *U. prolifera* shifted from strain I to strain II in the Southern Yellow Sea from 2008 to 2015, indicating that the annual dominant species of green tides in this region may vary. With the continuous rise in global temperatures and the increasingly frequent anthropogenic influences, the reproductive growth and stress resistance of *U. prolifera* is undergoing continuous changes, suggesting the likely existence of multiple strains within this species. However, limited research has been conducted on the actual existence of these strains and the differences between them.

In this study, ITS and 5S molecular marker technologies were employed to identify the dominant green tide species in two distinct years. Meanwhile, the gametophyte morphology of two *Ulva* strains, as well as the morphology and growth characteristics of their gamete-derived germlings, were systematically observed. Co-culture experiments of two *Ulva* strains (*QD-7* and *I08-1*) were then performed under natural light and temperature conditions. The morphology, growth patterns, and photosynthetic characteristics of the two strains were monitored to reveal the differences in their photosynthetic physiology across different years. The core objectives are to clarify *U. prolifera*’s specific reproductive mechanisms, elucidate the formation mechanisms of its ecotypes across different years, and provide a scientific basis for green tide prevention and rational resource utilization. The findings deepen the understanding of *U. prolifera*’s environmental adaptation, offer data for predicting its response to environmental changes, hold significant implications for formulating green tide control strategies and revealing bloom formation mechanisms, and lay a foundation for future research on *Ulva* interactions with other organisms and ecological control strategies.

## 2. Materials and Methods

### 2.1. Molecular Identification and Thallus Culture

In 2008, *U. prolifera* strains were collected from the sea surface at 36°2′18″ N, 120°21′33″ E along the coast of Qingdao, Shandong Province, China. In 2021, samples were collected from the sea surface at 36°3′48″ N, 120°23′34″ E in the same region. After the establishment of pure line thalli species, they were named *QD-7* and *I08-1*. Both strains were long-term preserved in the laboratory in the form of adult thalli. The preservation conditions were a temperature of 10 ± 0.5 °C, light intensity of 4 μmol photons·m^−2^·s^−1^, and a 12-h light/12-h dark photoperiod. Sterilized seawater supplemented with metal-enriched seawater medium (MES) nutrient solution was used as a culture medium [57,58].

Before the start of the culture experiment, *U. prolifera* was removed from the tissue cultivation bottle for molecular identification. Previous studies have described the ITS and 5S rDNA non-transcribed spacer sequences of the polymerase chain reaction (PCR) amplification reaction system in the molecular identification process [59,60]. The PCR products were detected by agarose gel electrophoresis and submitted to MAP Biotech Co., Ltd. (Shanghai, China) for sequencing. ITS and 5S rDNA primer sequences of the analyzed samples were obtained. The qualified sequence was copied to the Internal Center for Biotechnology Information database (https://www.ncbi.nlm.nih.gov/; accessed on 1 May 2025) for BLAST (Basic Local Alignment Search Tool), and a maximum likelihood (ML) phylogenetic tree was constructed using MEGA v11.0.13 (MEGA Limited, Auckland, New Zealand) software to identify the species. After the species were determined by molecular techniques, an appropriate number of thalli were cultured separately until they matured to form a gamete sac and released gametes.

### 2.2. Gamete Collection, Observation, and Cultivation

A small amount (~0.2 g) of adult thalli was transplanted into seawater-filled round glass bottles with a volume of 1 L aerated culture, and the light was set to 100–120 μmol photons·m^−2^·s^−1^. The other cultural conditions were the same as described above. Within 48 h, the thalli color changed from green to yellowish brown. The yellowish-brown thalli were removed and transferred into a 10 mL centrifuge tube. They were then rinsed two to three times with sterile seawater containing MES and placed in a culture environment. After a few minutes, the thalli began to release gametes, and the seawater became turbid. The gamete solution was pipetted with a rubber-head dropper to verify phototaxis and make temporary slides. The gamete morphology and flagella number were observed under a microscope, the gamete activity was confirmed, and the gamete size and flagella length were measured. The gamete solution was diluted with sterile seawater to an appropriate concentration (~200 per drop), and three drops of gamete solution were transplanted into seawater-filled round glass bottles with a volume of 250 mL of aerated culture. A total of three bottles were cultured; all of the culture medium was replaced after five days, and the culture was continued. After five days of culture, the young gametophytes were carefully scraped from the wall of the bottle, and 30 healthy and complete thalli were selected and transplanted into sterile seawater-filled round glass bottles with a volume of 1 L for aerating culture. The cultural conditions were the same as those described above.

### 2.3. Observation and Measurement of Gametophyte Morphology and Growth

**Figure 1:**
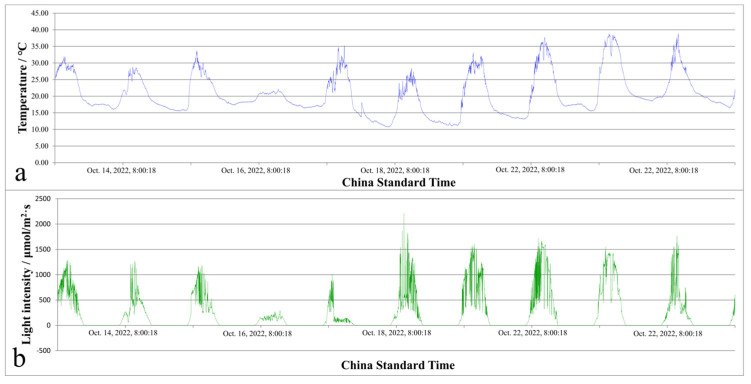
Natural temperature (**a**) and light intensity (**b**) variations.

Three thalli were randomly selected from each of the two 15-day-old *U. prolifera* strains, separately. After photographing and measuring the length and width of the main stem and the length of primary branches of the thalli, the thalli of the different strains were transferred into sterile seawater-filled round glass bottles with a volume of 1 L for aerating co-culture. One thallus of each strain was placed into a round glass bottle for co-culture. Three parallel experimental groups were set up and recorded as day 0. They were placed outdoors under natural light and temperature conditions for inflatable culture, and two light and temperature recorders, MX Temp/Light (ONSET Corporation, Cape Cod, MA, USA), were used to record ambient light and temperature changes (Figure 1a,b). Two rechargeable mobile aeratable pumps were alternately used for aeration. The thalli were photographed and measured every 48 h, and the culture medium was replaced with new sterile seawater containing MES. The above steps were repeated for 10 days. The specific growth rate (SGR) was calculated as follows [58,61,62]

(1) $$SGR=\frac{\left(InN-InN_{0}\right)}{t}$$

where N is the length (cm), width (cm), or wet weight (g) of the algae measured at a certain time; N_0_ is the length (cm), width (cm), or wet weight (g) of the algae measured at the previous time; and *t* (d) is the culture time.

### 2.4. Determination of Chlorophyll Fluorescence Parameters and Chloroplast Pigment Content

After being cultured for 10 days in the natural light and temperature environment, the algae were photographed, and their growth was measured. A 0.1 g sample of the thallus was taken and subjected to dark adaptation for 15 min. The chlorophyll fluorescence parameters of the algae were then measured using a dual-channel modulated chlorophyll fluorescence meter P700 & Chlorophyll Fluorescence Measuring System Dual-PAM-100 (Heinz Walz GmbH, Effeltrich, Germany). Among them, ETR, ETRmax, and YII were measured on thalli collected directly from natural light. Following this, a 0.1 g thallus sample was taken, and the chloroplast pigment was extracted with 95% alcohol. The ultraviolet–visible spectrophotometer SP-2500 (Shanghai Spectrometer Instrument Co., Ltd., Shanghai, China) was used to measure the values of *A*_470_, *A*_649_, and *A*_665_. The contents of chlorophyll *a* (Chl *a*), chlorophyll *b* (Chl *b*), and carotenoid (Car) were calculated by the following formulae [63]. Three parallel experiments were set up in each experimental group.

(2) $$C_{a}=13.95A_{665}-6.88A_{649}$$

(3) $$C_{b}=24.96A_{649}-7.32A_{665}$$

(4) $$C_{car}=\frac{1000A_{470}-2.05C_{a}-114.8C_{b}}{245}$$

where *C*_a_ and *C*_b_ represent the concentration of Chl *a* and Chl *b*, respectively; *C*_Car_ is the total concentration of Car; and *A*_470_, *A*_649_, and *A*_665_ are the absorbances of the chloroplast pigment extract at 470, 649, and 665 nm, respectively.

### 2.5. Statistical Analysis

Excel 2010 (Microsoft Corporation, Redmond, WA, USA) and SPSS 25 (SPSS Inc., Chicago, IL, USA) were used for statistical analysis, recording, editing, calculation, and mapping of the data, and one-way analysis of variance was performed. The statistical values were described as mean ± standard deviation. *p* < 0.05 was considered a significant difference between groups, and *p* < 0.01 was considered a highly significant difference between groups.

## 3. Results

### 3.1. Identification of Ulva prolifera Strains

**Figure 2:**
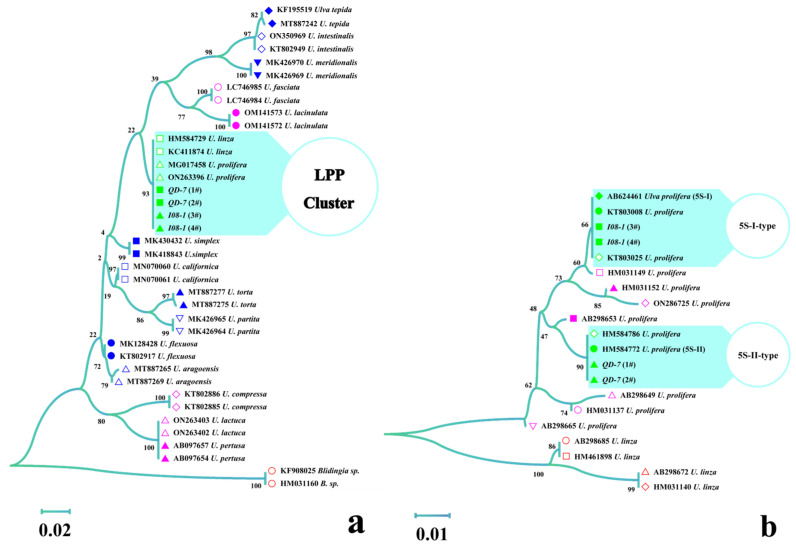
Phylogenetic analysis based on the maximum likelihood (ML) method. (**a**) Phylogenetic tree of the internal transcribed spacer (ITS) region from strains *QD-7* and *I08-1*; (**b**) phylogenetic tree of the 5S rDNA spacer from strains *QD-7* and *I08-1*.

In the ML phylogenetic tree based on ITS sequences, *QD-7* and *I08-1* were clustered with *U. linza* (HM584729 and KC411874) and *U. prolifera* (MG017458 and ON263396), and belonged to the *Ulva linza*-*procera*-*prolifera* (LPP) complex group (Figure 2a), indicating that the ITS region could not resolve the LPP complex group. Therefore, the 5S rDNA spacer was used to distinguish the LPP complex group. *QD-7* was clustered with 5S-II-type *U. prolifera* (HM584786 and HM584772), while *I08-1* was clustered with 5S-I-type *U. prolifera* (KT803025, KT803008, and AB624461), suggesting that *I08-1* represents 5S-I-type *U. prolifera* and *QD-7* represents 5S-II-type *U. prolifera* (Figure 2b).

### 3.2. Gamete Germling Morphology

**Figure 3:**
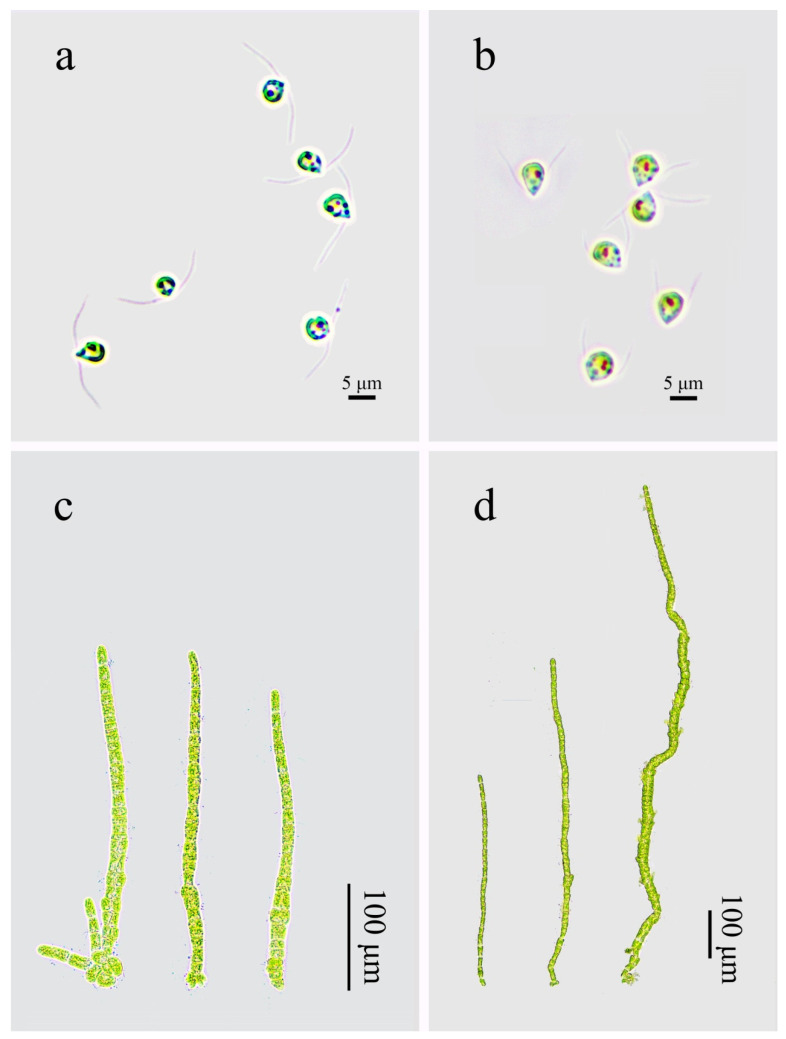
Morphology of gametes and germlings in *U. prolifera*. (**a**) Gametes of strain *QD-7*; (**b**) gametes of strain *I08-1*; (**c**) 6-day-old germlings of *QD-7*; (**d**) 6-day-old germlings of *I08-1*.

Gametes released from strains *QD-7* and *I08-1* were oval to pear-shaped, possessed two flagella, and exhibited positive phototaxis (Figure 3a,b). In 6-day-old germlings, the main axis grew linearly, with some cells developing lateral primary branches. A subset of *QD-7* individuals formed new branches originating from the holdfast region (Figure 3c,d).

**Figure 4:**
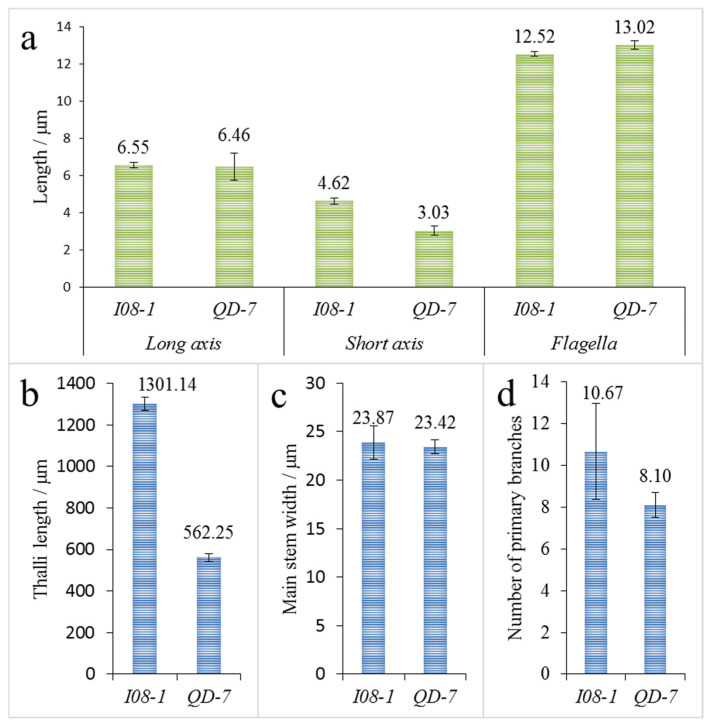
Gamete size, flagellar length, and growth of gamete germlings (*n* = 30). (**a**) Gamete size and flagellar length of the *QD-7* and *I08-1* strains; (**b**) germling length of 6-day-old *QD-7* and *I08-1* strains; (**c**) main stem width of 6-day-old *QD-7* and *I08-1* strains; (**d**) number of primary branches of 6-day-old *QD-7* and *I08-1* strains. Note: In (**a**), the numbers above histograms denote the average major axis length, minor axis length, and flagellum length of gametes for the two strains (units: μm). In (**b**–**d**), the numbers above histograms represent the average thalli length, main stem width, and primary branch count of thalli for the two strains (units: μm).

The gametes of *I08-1* were slightly larger than those of *QD-7*, and there was no significant difference in the long axis and flagellar lengths of gametes between the two strains. However, the short axis lengths were significantly different, with average values of 4.62 and 3.03 μm, respectively (Figure 4a). There was no significant difference in the main stem width of the gamete germlings of *I08-1* and *QD-7* strains at 6 days of age, with an average of 23.87 and 23.42 μm, respectively (Figure 4c). However, the thallus length showed a significant difference, with an average of 1301.14 μm and 562.25 μm, respectively (Figure 4b). This indicated that the gamete germlings of *I08-1* grew faster than those of *QD-7*. In addition, there was also a significant difference in the number of new branches on the main stem of the two strains of gamete germlings (one cell bulge, Figure 3c,d), with an average of 10.67 and 8.10 branches per germling, respectively (Figure 4d).

### 3.3. Adult Thalli Morphology

**Figure 5:**
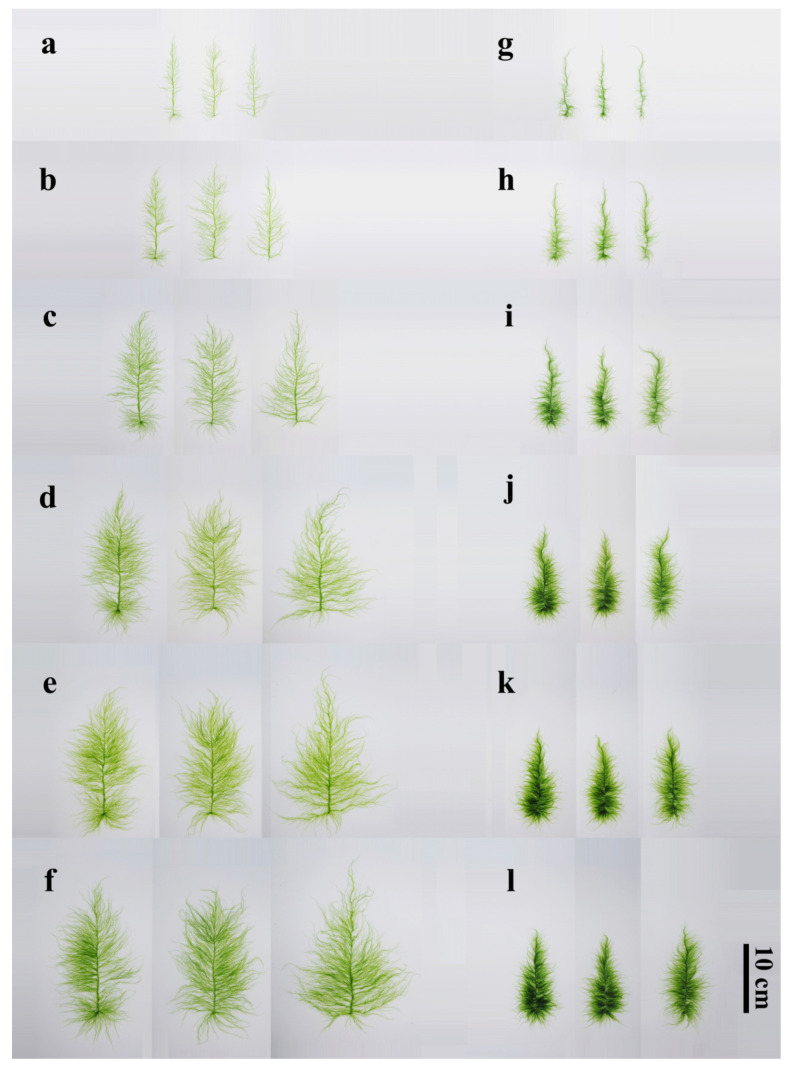
Morphological photographs of 15-day-old *QD-7* and *I08-1* co-cultured for 2, 4, 6, 8, and 10 days under natural light and temperature conditions. (**a**,**g**) Thalli after 15 days of culture at 20 °C with gametes of the *QD-7* and *I08-1* strains. (**b**–**f**) Morphology of the gametophytes of the *QD-7* strain cultured for 15 days at 20 °C, and then cultured for 2, 4, 6, 8, and 10 days under natural light and temperature conditions. (**h**–**l**) Gametes of the *I08-1* strain were cultured for 15 days at 20 °C and then cultured under natural light and temperature conditions for 2, 4, 6, 8, and 10 days. Note: Two biological replicates were performed within the same season, each consisting of three technical replicates. Results showed high consistency between replicates.

**Figure 6:**
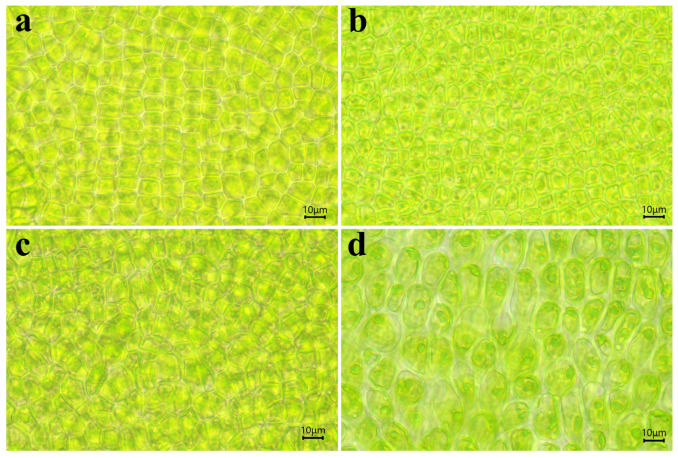
Morphology of the central branch cells of the main branch in *QD-7* and *I08-1* strains before and after co-culture. (**a**,**c**) Morphology of central branch cells in the main branch of *QD-7* and *I08-1* strains, respectively, cultured at 20 °C for 15 days (corresponding to the thalli in Figure 5a,g). (**b**,**d**) Morphology of central branch cells in the main branch of *QD-7* and *I08-1* strains, respectively, after being cultured at 20 °C for 15 days, followed by 10 days under ambient light and temperature conditions (corresponding to the thalli in Figure 5f,l).

After the gametes of *QD-7* and *I08-1* strains were cultured for 15 days at 20 °C, the thalli developed into a “sapling” morphology, with *I08-1* appearing greener than *QD-7*. The thalli of 15-day-old *QD-7* were emerald green, with third-order branches. The base of the main stem exhibited distinct holdfasts and well-defined branching. The mid-region of the main stem was flattened and broad, while the basal portion gradually narrowed into a dark green, hollow, tubular structure. The main stem of the tip was not obvious, and the longer primary branch on the main stem was unevenly and sparsely distributed, with more small branches (Figure 5a). The main branch middle part cell surface view is square or polygonal in shape, with a relatively regular cell arrangement, appearing yellow-green. The cell contains one nucleus and a flake-like chromatophore located on the protoplast surface, along with 1 or 2 starch nuclei (Figure 6a). The thalli of *I08-1* were dark green, the main branch comprised an irregular cylindrical tube, the base had an obvious holdfast, and the branches near the base were dense and elongated. From the base to the apex, primary branches progressively shortened, and the slender apical region was prone to curling. The main branch was prominent, with primary branches densely arranged along the stem, creating a hairy appearance. These branches frequently intertwined with each other or coiled around the main stem under moving seawater. The thalli also developed second-order branches (Figure 5g). The cells in the middle part of the main branch have a polygonal shape when viewed from the surface. They vary in size and are arranged irregularly. The cells are green in color. Inside each cell, there is one nucleus and a flake-shaped chromatophore located on the surface of the protoplast. Additionally, the cells contain 1 or 2 starch nuclei (Figure 6c).

With the increase in the culturing duration, the thalli of both strains gradually grew upwards. The thalli of *QD-7* were yellow-green and cypress-shaped. The main stem was flat and wrinkled, and the longer primary branch became longer. The inward contraction of the base of some branches and the junction of the main stem became smaller and were easier to detach from the main stem, which was consistent with the morphology of floating *U. prolifera* in the sea area. The branch level was 4–5. The base of the branch growing on the base holdfast had a rhizoid, which was easy to detach from the main stem to become a new individual. There were many small branches on the main stem that could become a new, longer primary branch (Figure 5b–f). After 10 days of cultivation under natural light and temperature conditions, the cells in the middle part of the main branch of the alga also appeared yellow-green when viewed from the surface. Compared to before the co-culture, the cells became smaller, with obvious cell gaps. The cells were yellow-green in color. Inside each cell, there was one nucleus and a flask-shaped chromatophore located on the surface of the protoplast. Additionally, the cells contained 1 or more starch nuclei (Figure 6a,b). In *I08-1*, the thalli were cypress-shaped and dark green, the branches on the main stem became dense and long, almost completely wrapping the main stem, and the branch level was 2–3. Due to the dense distribution of small branches on the main stem, the main stem began to become rhizoid (Figure 5h–l). After 10 days of cultivation under natural light and temperature conditions, the cells in the middle part of the main branch of the alga appeared long-elliptical in shape when viewed from the surface, with their contours approximating an arc-like shape. They transformed into rhizoid-like cells and became significantly larger than before the co-culture. Inside each cell, there was one nucleus and a flask-shaped chromatophore located on the surface of the protoplast. Additionally, the cells contained one or more starch nuclei (Figure 6c,d). The results showed that the morphology of *QD-7* was more adaptable to the environment and that it grew faster compared with *I08-1*.

### 3.4. Adult Thalli Growth

**Figure 7:**
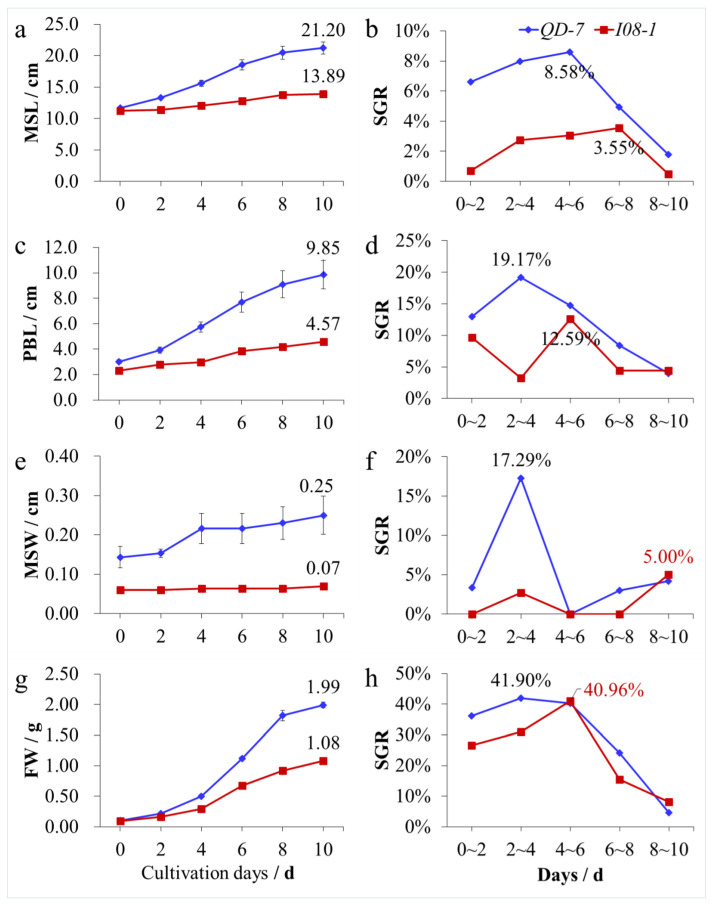
Growth of adult thalli. (**a**) Changes in the length of the main stem (MSL); (**b**) specific growth rate (SGR) variation in *U. prolifera* length; (**c**) changes in the length of primary branches (PBL); (**d**) SGR variation in PBL; (**e**) changes in the width of the main stem (MSW); (**f**) changes in the SGR of the MSW; (**g**) change in fresh weight (FW); and (**h**) SGR of FW. Note: In (**a**), numbers above the line show the average MSL (cm) of the two strains after 10 days of culture; in (**b**), numbers adjacent to the line indicate the maximum SGR of MSL within 10 days; in (**c**), numbers above the line denote the average PBL (cm) after 10 days; in (**d**), numbers next to the line represent the maximum SGR of PBL within 10 days; in (**e**), numbers above the line show the average MSW (cm) after 10 days; in (**f**), numbers close to the line are the maximum SGR of MSW within 10 days; in (**g**), numbers above the line reflect the average FW of thalli after 10 days; in (**h**), numbers adjacent to the line indicate the maximum SGR of thalli FW within 10 days.

Under natural light and temperature conditions, with an increasing number of culture days, the main stem length (Figure 7a), primary branch length (Figure 7c), main stem width (Figure 7e), and fresh weight (Figure 7g) of the *QD-7* and *I08-1* strains increased, but the increases in the *QD-7* strain were significantly greater than those of *I08-1*. The width change in the two *U. prolifera* strains was not very large, and the width change in *I08-1* was the least obvious (Figure 7e). The young thalli of 15-day-old *QD-7* and *I08-1* were cultured for another 10 days. The main stem lengths of *QD-7* and *I08-1* were 21.20 and 13.89 cm (Figure 7a), the primary branch lengths were 9.85 and 4.57 cm (Figure 7c), the main stem widths were 0.25 and 0.07 cm (Figure 7e), and the fresh weights were 1.99 and 1.08 g per thallus, respectively (Figure 7g). In addition, in the 10-day co-culture, except for the SGR of the primary branch length, main stem width, and fresh weight of *I08-1* at 8–10 days, and the SGR of fresh weight at 4–6 days being slightly higher than that of *QD-7*, the SGR of the main stem length, width, primary branch length, and fresh weight of *QD-7* was generally higher than that of *I08-1*. During the 10-day co-culture, the maximum SGRs of the main stem length and width, primary branch length, and fresh weight of *QD-7* and *I08-1* were 8.58% and 3.55% (Figure 7b), 19.17% and 12.59% (Figure 7d), 17.29% and 5.00% (Figure 7f), and 41.90% and 40.96% (Figure 7h), respectively. These results showed that under natural light and temperature conditions, the 15-day-old *QD-7* strain grew significantly faster than the *I08-1* strain.

### 3.5. Chlorophyll Fluorescence Parameters

**Figure 8:**
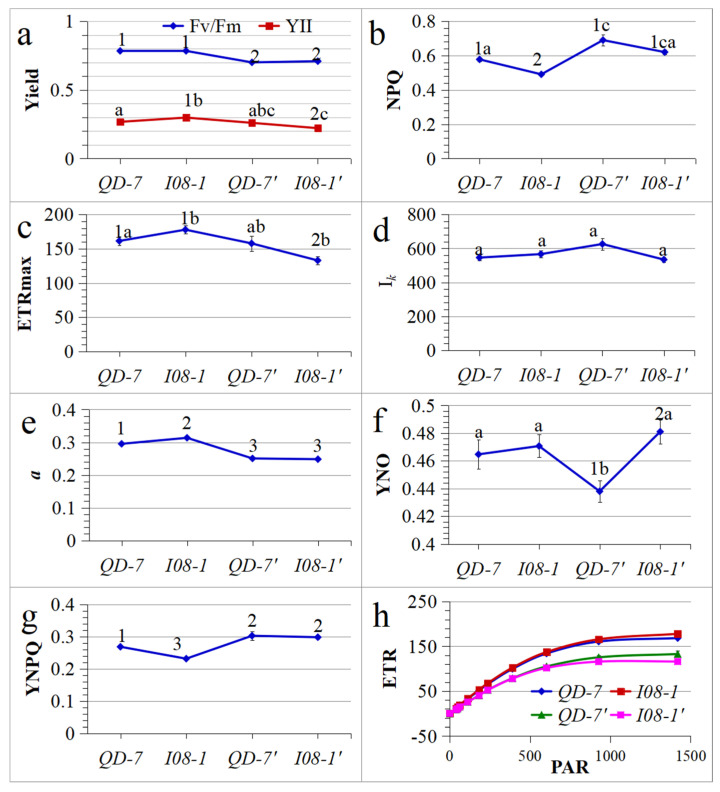
Chlorophyll fluorescence parameters of 15-day-old gametophytes; 15-day-old gametophytes were further cultured for another 10 days under natural light and temperature conditions. (**a**) Maximum photochemical quantum yield (F_v_/F_m_) and actual photochemical quantum yield (YII); (**b**) non-photochemical fluorescence quenching (NPQ); (**c**) maximum electron transfer rate (ETRmax); (**d**) minimum saturated irradiance (I*_k_*); (**e**) initial slope of the fast light curve related to the quantum efficiency of photosynthesis (*a*); (**f**) quantum yield of PSII in non-regulated energy dissipation (YNO); (**g**) quantum yield of PSII in regulated energy dissipation (YNPQ); (**h**) relative electron transfer rate (ETR) fitting curve. Three parallels (*n* = 3) were set in each experimental group. *QD-7* and *I08-1* were the gametophytes of two strains of *U. prolifera* cultured for 15 days at 20 °C. *QD-7’* and *I08-1’* were gametophytes of two *U. prolifera* strains cultured for 15 days at 20 °C and then co-cultured for 10 days under natural light and temperature conditions. The different letters and numbers above the line chart indicate that the chlorophyll fluorescence parameters of the two strains were significantly (*p* < 0.05) and highly significantly (*p* < 0.01) different before and after co-culture. The same letter or the same Arabic number indicates no significant difference.

Before the co-culture experiment, the initial slope of the fast light curve related to the quantum efficiency of photosynthesis (*a*), quantum yield of PSII in regulated energy dissipation (YNPQ), and the non-photochemical fluorescence quenching (NPQ) between the two strains (*QD-7* and *I08-1*) showed highly significant differences. The maximum electron transfer rate (ETRmax) and actual photochemical quantum yield (YII) showed significant differences, while the minimum saturated irradiance (I*_k_*) and maximum photochemical quantum yield (F_v_/F_m_) showed no significant difference between strains. After co-culture, only the quantum yield of PSII in non-regulated energy dissipation (YNO) showed a highly significant difference between strains, whereas F_v_/F_m_, YII, NPQ, ETRmax, I*_k_*, *a*, and YNPQ showed no significant difference (Figure 8a–g). Before and after co-culture, the F_v_/F_m_, YNPQ, and *a* of *QD-7* showed highly significant differences compared with *I08-1*; the NPQ and YNO showed significant differences, while there were no significant differences in YII, ETRmax, and I*_k_*. Before and after co-culture, the F_v_/F_m_, YII, NPQ, *a*, and YNPQ of *I08-1* showed highly significant differences, while ETRmax, I*_k_*, and YNO showed no significant differences (Figure 8a–g). Compared with *QD-7* before co-culture, the F_v_/F_m_, ETRmax, *a*, and YNPQ of *I08-1* after co-culture were highly significantly different; YII was significantly different; and NPQ, I*_k_*, and YNO were not significantly different. Compared with *I08-1* before co-culture, the F_v_/F_m_, NPQ, *a*, and YNPQ of *QD-7* after co-culture were highly significantly different; YNO was significantly different, while YII, ETRmax, and I*_k_* were not significantly different (Figure 8a–g).

Furthermore, before co-culture, the ETR of *I08-1* was higher than that of *QD-7*. After co-culture, however, the ETR of *QD-7’* became higher than that of *I08-1’*. Notably, the ETR values of both strains (*QD-7’* and *I08-1’*) after co-culture were lower than their respective values before co-culture (Figure 8h). These results revealed that the chlorophyll fluorescence parameters of *QD-7* and *I08-1* show significant differences under natural light and temperature conditions, indicating significant differences in photosynthesis between the two.

### 3.6. Chloroplast Pigment Content

**Figure 9:**
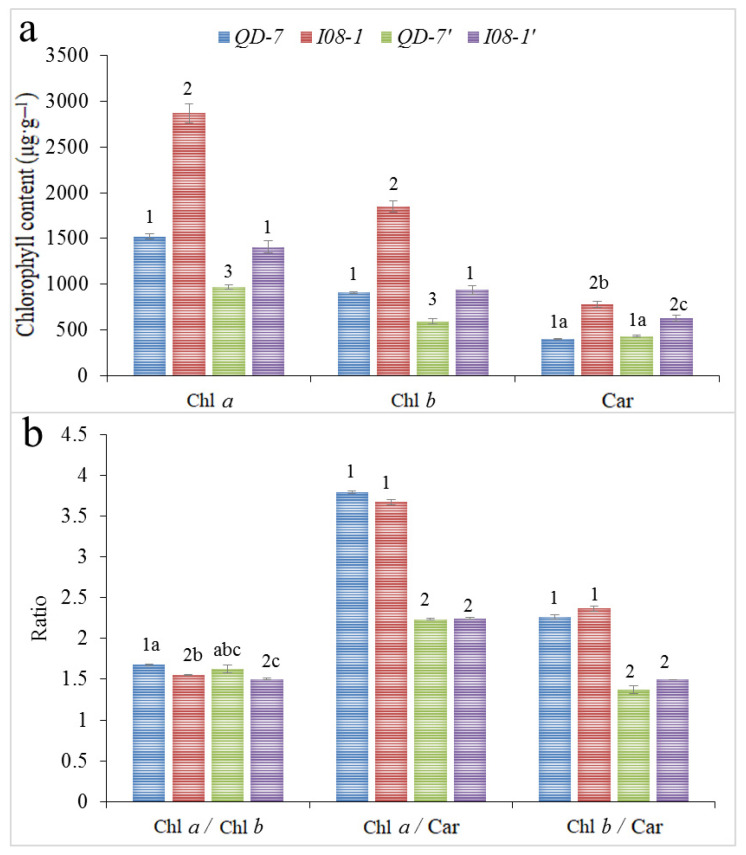
Chloroplast pigment contents (**a**) and their content ratios (**b**) of 15-day-old gametophytes (*QD-7* and *I08-1*) cultured at 20 °C, as well as those of gametophytes (*QD-7’* and *I08-1’*) after an additional 10-day cultivation under natural light and temperature conditions. Different letters and numbers above the histogram indicate a significant (*p* < 0.05) and highly significant (*p* < 0.01) difference in chloroplast pigment content and its ratio before and after co-culture between *QD-7* and *I08-1*. The same letter or the same Arabic number indicates no significant difference.

Chl *a* and Chl *b* contents of both *QD-7* and *I08-1* showed highly significant differences before and after co-culture. The Car content of *I08-1* showed significant differences before and after co-culture, but there was no significant difference in Car before and after the co-culture of *QD-7*. Before co-culture, the contents of Chl *a*, Chl *b*, and Car highly significantly differed between strains. After co-culture, the contents of Chl *a*, Chl *b*, and Car in the two strains were also highly significantly different. Comparing *QD-7* before co-culture with *I08-1* after co-culture, there was no significant difference in the contents of Chl *a* and Chl *b*; however, Car showed a highly significant difference. Comparing *I08-1* before co-culture with *QD-7* after co-culture, the contents of Chl *a*, Chl *b*, and Car showed highly significant differences (Figure 9a).

For *QD-7* and *I08-1* before co-culture, only Chl *a*/Chl *b* significantly differed, whereas there was no significant difference in Chl *a*/Car or Chl *b*/Car. After co-culture, there was no significant difference in Chl *a*/Chl *b*, Chl *a*/Car, or Chl *b*/Car. Comparing before and after co-culture, the Chl *a*/Car and Chl *b*/Car of both *QD-7* and *I08-1* showed highly significant differences. The Chl *a*/Chl *b* of *I08-1* showed significant differences, but there was no significant difference in the Chl *a*/Chl *b* of *QD-7*. Compared with *QD-7* before co-culture and *I08-1* after co-culture, Chl *a*/Chl *b*, Chl *a*/Car, and Chl *b*/Car showed highly significant differences. Compared with *I08-1* before co-culture and *QD-7* after co-culture, Chl *a*/Car and Chl *b*/Car also showed highly significant differences, but there was no significant difference in Chl *a*/Chl *b* (Figure 9b).

The chloroplast pigment content of *I08-1* was highly significantly higher than that of *QD-7* before and after co-culture. Except for Chl *a*/Chl *b* before the co-culture of *QD-7* being significantly higher than that of *I08-1*, the ratios of the other chloroplast pigment contents were not significantly different. The physiological mechanism of *I08-1* was a greener color than *QD-7* and that of *QD-7* was a faster growth rate.

## 4. Discussion

*U*. *prolifera* blooms, exhibiting opportunistic dynamics and strong adaptability, are a worldwide ecological issue, predominantly occurring in eutrophic coastal regions. With the advancement of industrialization, and the intensification of global climate change and marine eutrophication, green tides have become a regular occurrence in coastal areas worldwide, including China [60,64,65], the USA [66,67,68], Japan [69], France [67], South Korea [70], Australia [71], the Philippines [72], and South Africa [73], among others, and are generally located in eutrophic waters. The species involved in green tide outbreaks differ among countries, but these events are mainly caused by *Ulva* green algae. These blooms consistently exhibit the characteristic dynamics of opportunistic macroalgae: rapid biomass accumulation under favorable nutrient and light conditions, followed by mass dispersal or mortality triggered by physical disturbances (e.g., storms, low tides, currents) or self-shading. The recurrent nature of these events across geographically distinct systems underscores *U. prolifera*’s inherent ecological strategy as a highly adaptable, opportunistic species capable of exploiting nutrient-enriched coastal habitats worldwide. In China, the main species are *U. prolifera* and *U. meridionalis*. There are many reasons for the outbreak of green tides, but it mainly involves environmental factors such as seawater eutrophication, light, temperature, salinity, and the biological characteristics of green tide algae. This also involves the genetic variation of *U*. *prolifera* populations, interspecific competition, and their interactions with microbial communities. For instance, the metabolic synergy between *U*. *prolifera* and symbiotic bacteria may further drive its population to bloom by promoting nutrient cycling or enhancing stress resistance. The core contradiction behind the long-term outbreak of the *U. prolifera* green tide in China’s Yellow Sea lies in its unique floating migration mechanism. Originating mainly from aquaculture areas in Jiangsu, *U. prolifera* continues to proliferate in open waters as it migrates with ocean currents. Unlike typical substrate-attached worldwide green tides (such as those in Brittany), this dynamic migration characteristic weakens the effectiveness of local governance and forms a two-stage “open-sea carbon sink/nearshore disaster” pattern through the coupling of terrestrial nutrients and ocean currents (open seas have carbon sequestration potential, while onshore landing may cause ecological degradation). Current strategies mainly mitigate economic losses, but fundamental governance requires coordinated breakthroughs in land–sea bottlenecks: integrating source regulation, path interception, and high-value conversion to build a cross-regional prevention and control system.

In this study, the morphology, growth, and photosynthetic characteristics of floating *Ulva* in 2008 and 2021 were studied under natural light and temperature conditions. Due to the morphological similarity of *Ulva*, molecular marker technology plays an important role in the identification of green algae species. The identification and analysis of ITS and 5S molecular marker technology showed that *I08-1* was 5S-I type and *QD-7* was 5S-II type *U. prolifera*. Morphological observations showed that the *I08-1* strain is dark green and cypress-like, with dense two- to three-level small branches on the main branches. Branches shading each other may cut light-gathering efficiency, intensify intraspecific competition, and slow growth (Figure 5g–l and Figure 7a–h). In contrast, the *QD-7* strain has a yellow-green thallus with a flat and wrinkled main branch. Its four- to five-level branches are alternately distributed, creating larger gaps between branches. This structure not only enhances light-capturing efficiency but also increases the surface area, boosting the absorption of nutrients like nitrogen and phosphorus (Figure 5a–f and Figure 7a–h). Further microscopic observations revealed that the *QD-7* thallus surface has many cellular protuberances resembling “buds”. This may relate to its efficient material exchange and strong vegetative propagation ability.

Our study shows that *QD-7* exhibits a more environmentally adaptive morphology, characterized by stronger photosynthetic capacity and faster growth rates. Its unique four- to five-level branching structure and high morphological plasticity likely enhance environmental adaptability. The yellow-green pigmentation improves light energy utilization efficiency. The detachment-prone branch bases (Figure 5b–f) facilitate efficient fragmentation-based reproduction: detached segments (including rhizoids) rapidly develop into new individuals—a trait well-suited to the marine floating niche. Unlike *I08-1*, where dense branching leads to energy dissipation and the main branch algal cells resemble the basal cells of the algal body, as if about to transform into rhizoids. (Figure 5h–l), *QD-7*’s sparse main branches minimize frictional damage from water currents. Furthermore, its capacity for small branches to transform into new main branches enables dynamic resource allocation toward highly photosynthetic structures. The hollow tubular basal structure provides dual functionality: anchoring stability and nutrient storage. In contrast, *I08-1*’s dense branching compromises physiological efficiency. These features confer three key advantages on *QD-7* in disturbed environments: (1) fragmentation-driven exponential population expansion, (2) environmental fluctuation buffering through branch redundancy, and (3) rapid algal mat formation via surface coverage. This combination underpins *QD-7*’s ecological dominance in Yellow Sea green tides.

Photosynthesis is one of the most important metabolic activities in plants [74]. The F_v_/F_m_ represents a theoretical value of the photosynthetic potential of plants and algae, indicating their physiological status [75,76,77,78,79]. Under normal conditions, this parameter generally changes very little and is unaffected by species and growth conditions. However, when plants are subjected to stress, the value of F_v_/F_m_ decreases rapidly, indicating that the efficiency of PSII photochemical conversion decreases. F_v_/F_m_ is an important indicator that has been widely used to determine the magnitude of stress on algae [78]. *Ulva*’s highly efficient photosynthetic properties allow it to thrive worldwide [80]. In this study, the F_v_/F_m_ experimental values of *U. prolifera* changed significantly compared with the initial values, suggesting that *U. prolifera* was stressed by high temperature and high light intensity under natural light and temperature conditions. The F_v_/F_m_ value, which characterizes the maximum photochemical efficiency of PSII, decreased significantly under high temperature and light intensity but remained around 0.7 for both *Ulva* strains. This indicates that *Ulva* effectively alleviated photosystem damage by activating the NPQ mechanism and antioxidant enzymes like superoxide dismutase (SOD). Notably, the YNPQ value of the *QD-7* strain was higher than that of the *I08-1* strain before and after exposure to high temperature and light intensity, suggesting a more active photoprotection mechanism in *QD-7*. The effective (actual) light quantum yield YII is also used as an important indicator to evaluate the changes in the photosynthetic activity of algae in different environments [81]. After co-culture under natural high temperature and light intensity, the YII of the two strains of *U. prolifera* decreased slightly, but there was no significant change, indicating that the YII of the thallus could typically be maintained at a relatively stable level under high temperature and high light stress. *U*. *prolifera* was in good health and maintained good photosynthetic activity under high temperatures and high light intensity.

The quantum yield of regulatory energy dissipation at PSII (YNPQ) is an important indicator of photoprotective capacity. After the co-culture experiment, the YNPQ of both strains increased, suggesting that *U. prolifera* likely received excessive light energy. This observation implies that *U. prolifera* could protect itself through regulated thermal dissipation, demonstrating a possible high-light protection mechanism. YNO represents the quantum yield of non-regulated energy dissipation at PSII, serving as an indicator of photodamage susceptibility. The YNO values of *QD-7* and *I08-1* showed marked divergence after co-culture: *QD-7* exhibited a significant decrease, while *I08-1* displayed a substantial increase. These differential responses suggest that the photoconversion and photoprotective regulation mechanisms in *QD-7* (e.g., thermal dissipation) could effectively process the absorbed light energy, potentially minimizing uncontrolled energy dissipation. However, for *I08-1*, the incident light intensity likely exceeded its acceptable level, and the algae may have been damaged. The findings suggest that *QD-7* likely possesses enhanced photoprotective mechanisms relative to *I08-1*, potentially conferring greater adaptability to high-light environments.

The relative ETR of plants can reflect their photosynthetic efficiency and the photoprotective effect of dissipating excess light energy [82]. As photosynthetically active radiation (PAR) increased, the ETR typically showed a corresponding rise, suggesting that *U. prolifera* exhibits adaptability to high-light conditions. I*_k_* can reflect the ability of algae to tolerate high light [83]. High-light-tolerant plants usually have higher maximum photosynthetic rates [84]. An increase in algae light intensity is the main reason for a reduction in photosystem activity [85]. The ETRmax of *I08-1* exhibited greater variation than that of *QD-7* post-treatment, with significantly lower absolute values observed in *I08-1*. Nevertheless, both strains maintained elevated ETRmax levels (Figure 8c). After exposure to natural high light and temperature stress, the *QD-7* strain exhibited higher ETRmax and I*_k_* values than *I08-1*. These observations suggest that the photosynthetic apparatus of *QD-7* could maintain enhanced electron transport efficiency under high-light conditions. The *a* of the two strains was also highly significantly different, but both remained at a high level (Figure 8e). This suggests that *U. prolifera* may have a strong light-harvesting ability, which could be an important factor in its becoming a dominant green tide species.

Following co-culture, the light response curves of both *U. prolifera* strains exhibited downward shifts in ETR, with *I08-1* showing more pronounced suppression. Notably, prior to and post co-culture, ETR in both *QD-7* and *I08-1* demonstrated progressive increases with rising photosynthetically active radiation (PAR). These patterns suggest that the strains possess substantial adaptive capacity to natural high-temperature and high-light conditions, potentially associated with *U. prolifera*’s photosynthetic pigment content. Corresponding patterns were observed between ETR dynamics and changes in photosynthetic pigments (Chl *a*, Chl *b*, and Car) in both strains (Figure 8h and Figure 9a).

The content of photosynthetic pigments in chloroplasts is considered to be one of the important indicators of plant photosynthetic performance. It mainly affects the absorption, transmission, and transformation of light energy, and regulates the conversion and distribution of NADPH and ATP between PSII and PSI, thereby affecting plant photosynthetic efficiency [78,86]. The content of Chl *a* in plants is not only related to the cyclic electron transport of PSI and the synthesis of ATP in cyclic photosynthetic phosphorylation, but also to the transfer rate of *e*^-^ and *H*^+^ and the reduction in NADP^+^ in non-cyclic photosynthetic phosphorylation. Chl *b* is related to the absorption of light energy by chloroplasts and may affect the conversion of light energy into chemical energy by plants, thus affecting ATP synthesis. The Car performs two major functions: light energy capture and light damage defense. In algal photosynthesis, carotenoids protect chlorophyll from oxidation of active substances caused by radiation, and their content is related to the protective ability of the plant photosynthetic system itself [87]. Carotenoids assist in light harvesting and directly protect against photodamage through the xanthophyll cycle, such as via zeaxanthin synthesis. It appears that high chlorophyll content is indicative of a high light-harvesting complex content in PSII. The vegetative cells of *U. prolifera* contained multiple chloroplasts, the specific site of photosynthesis. The chlorophyll contents of *QD-7* and *I08-1* before and after co-culture exceeded 590 μg/g (Figure 9a). These findings suggest that both strains exhibit robust capabilities for light energy capture, conversion, and photoprotection. This adaptive competence likely stems from *U. prolifera*’s long-term evolutionary adaptation to intertidal environments characterized by high temperature and high-light exposure. Beyond its roles in light absorption and energy transfer, Car helps protect *U*. *prolifera* chlorophyll from photooxidation. Notably, chlorophyll and Car contents in both strains were generally maintained at elevated levels throughout co-culture (Figure 9a). Under high-light conditions, Car mitigated chlorophyll photooxidation, thereby contributing to photosystem protection and supporting sustained photosynthetic efficiency.

The NPQ can reflect the light protection ability of *U. prolifera* and its ability to safely dissipate excess light energy into heat energy [88]. It appears that the NPQ values of *QD-7* and *I08-1* were higher than those before the co-culture experiment, suggesting an increase in heat dissipation and excess light energy (Figure 8b). The Chl *a*/Chl *b* ratio may be functionally linked to the modulation of NPQ through xanthophyll cycle-dependent energy dissipation mechanisms [89]. Also, this suggests that the dynamic distribution of chloroplasts in *Ulva* cells, like their migration to the cell periphery under high light, is an important strategy to reduce photodamage. In this study, NPQ in *U. prolifera* increased as Chl *a*/Chl *b* decreased (Figure 8b and Figure 9b), indicating likely enhanced photoprotective capacity in this species. The dynamics of Chl *a*/Chl *b* ratios in *U. prolifera* may reflect coactions between genetic constraints and phenotypic plasticity (Figure 9b). Pre-culture differences between strains *QD-7* and *I08-1* suggest heritable variation in photosynthetic apparatus configuration, while post-culture ratio shifts appear correlated with NPQ and light-response adjustments under high-temperature/light stress. As a dominant green-tide species, *U. prolifera* can modulate its Chl *a*/Chl *b* ratio to potentially optimize light-energy capture and photoprotection in fluctuating intertidal environments. Such plasticity could represent an evolutionary adaptation strategy facilitating photosynthetic optimization under variable light conditions. Collectively, these findings support viewing Chl *a*/Chl *b* as a phenotype of genotype-environment interaction (G × E) within complex photoadaptive mechanisms, warranting further investigation.

## 5. Conclusions

In summary, this study observed the gametophyte morphology and size of the *QD-7* and *I08-1* strains and the morphology and growth of gamete-germlings in the laboratory. The growth of gamete-germlings of *I08-1* was faster than that of *QD-7*. Following this, the 15-day-old gametophytes of the *QD-7* and *I08-1* strains were co-cultured under naturally high temperatures and high light intensity. The morphology, growth, and photosynthetic activity of the two strains were observed. The morphology of the adult algae of *QD-7* was more adaptable to the environment, and it grew faster compared with *I08-1*. In the natural light and temperature environment, the chlorophyll fluorescence parameters of *QD-7* and *I08-1* showed significant differences, revealing that there were significant differences in photosynthetic efficiency between the two strains. By measuring the chlorophyll fluorescence parameters and chloroplast pigment content of the two *U. prolifera* strains, the algae were shown to be obviously stressed by high temperature and light intensity under natural light and temperature conditions. However, *U. prolifera* had a good self-protection and adaptation mechanism to high temperature and light, was not severely damaged, and still maintained a high photosynthetic efficiency. The experimental values based on the YNO and ETRmax showed that *QD-7* had a stronger high-light protection mechanism and photosynthetic capacity than *I08-1*, and could better adapt to a high-light environment. These conclusions enhance our understanding of the environmental adaptation mechanism of *U. prolifera* and reveal differences in morphology, growth, and photosynthesis of the two *U. prolifera* strains. This is helpful for predicting the response of *U. prolifera* to changing environments and can provide scientific data reference for the formulation of green tide prevention and control strategies. It also advances the understanding of *U. prolifera* green tide formation mechanisms.

Overall, this study highlights significant differences in *U*. *prolifera* strains in morphology, photosynthesis, and environmental adaptability, offering a foundation for predicting green tide dynamics. Future studies should employ metagenomic approaches to investigate the *Ulva*–microbiome interaction network and develop ecological control strategies through allelopathic substances or microbial competition. Additionally, given the high-light adaptability of the 5S-II-type *Ulva*, enhanced satellite-based monitoring is needed to mitigate ecological risks posed by green tides.

## Data Availability

The original contributions presented in this study are included in the article, and further inquiries can be directed to the corresponding author.
